# Recent advances in graphene family materials toxicity investigations

**DOI:** 10.1007/s11051-012-1320-8

**Published:** 2012-11-29

**Authors:** Agnieszka Maria Jastrzębska, Patrycja Kurtycz, Andrzej Roman Olszyna

**Affiliations:** Faculty of Materials Science and Engineering, Warsaw University of Technology, Woloska 141, 02-507 Warsaw, Poland

**Keywords:** Graphene family materials, Graphene, Graphene oxide, Reduced graphene, Graphite, Toxicity, In vitro, In vivo, Mechanisms, Functionalization, Bacteria, Mammalian cells, Animals

## Abstract

Recently, graphene family materials (GFMs) have been introduced among all fields of science and still get numerous attention. Also, the applicability of these materials in many areas makes them very attractive. GFMs have attracted both academic and industrial interest as they can produce a dramatic improvement in materials properties at very low filler content. This article presents recent findings on GFMs toxicity properties based on the most current literature. This article studies the effects of GFMs on bacteria, mammalian cells, animals, and plants. This article also reviews in vitro and in vivo test results as well as potential anticancer activity and toxicity mechanisms of GFMs. The effect of functionalization of graphene on pacifying its strong interactions with cells and associated toxic effects was also analyzed. The authors of the article believe that further work should focus on in vitro and in vivo studies on possible interactions between GFMs and different living systems. Further research should also focus on decreasing GFMs toxicity, which still poses a great challenge for in vivo biomedical applications. Consequently, the potential impact of graphene and its derivatives on humans and environmental health is a matter of academic interest. However, potential hazards sufficient for risk assessment first need to be investigated.

## Introduction

**Fig. 1:**
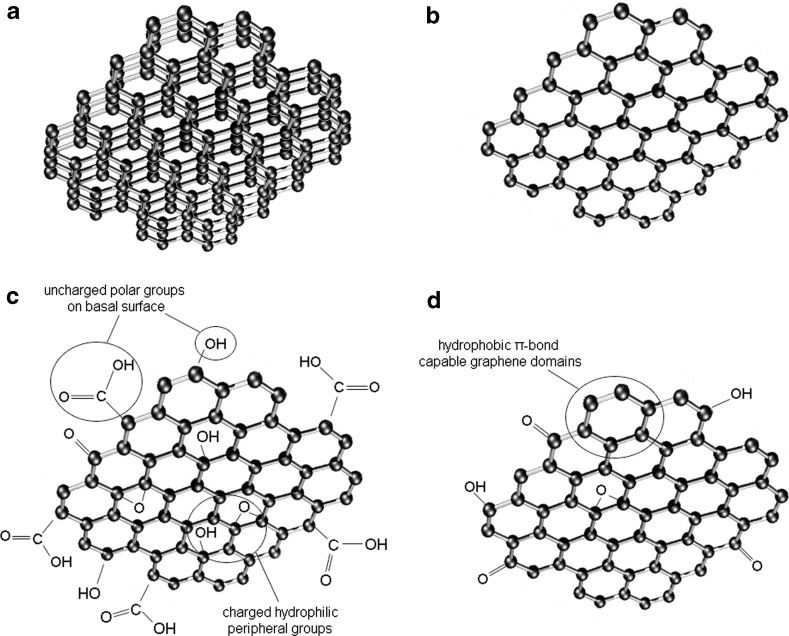
The members of the graphene family materials: few-layered graphene (**a**), graphene nanosheet (**b**), graphene oxide (**c**), and reduced graphene (**d**)

Graphene is a newly emerging member of carbon materials with sp2-hybridized single-atom-layer structure. It is a typical two-dimensional material made of carbon atoms packed densely in a honeycomb crystal lattice. Graphene is believed to be composed of benzene rings stripped of their hydrogen atoms (Neto and Peres 2006). In 2004, Geim and coworkers successfully identified single layers of graphene and other two-dimensional crystals (Novoselov et al. 2004). Related materials include few-layer graphene, graphene nanosheets, graphene oxide, and reduced graphene oxide and can be included in graphene family materials (GFM) (Sanchez et al. 2011) (Fig. 1).

Graphene family materials have drawn much scientific attention and technological interest since their discovery due to their unique electronic and mechanical properties, specific magnetism, excellent mobility of charge carriers, and high thermal conductivity (Neto et al. 2009). High surface area, excellent conductivity, outstanding mechanical strength, and extraordinary electrocatalytic activities of these materials have also been reported in the literature (Zhang et al. 2011).

Graphene and the related materials demonstrate great potential for applications in many areas, such as field effect transistors, solar cells, sensors, and adsorbent for heavy metal removal (Li et al. 2011), lithium ion batteries, solar cells, and electrochemical super capacitors (Zhang et al. 2011).

Graphene and graphene oxide layers have also been examined in relation to building new composite materials (Wang et al. 2011). These novel nanocomposite materials have great potential for application, such as constructing electrochemical devices, energy storage devices as well as catalysts (Wang et al. 2011).

Recent studies have shown that graphene and graphene oxide exhibit several unique modes of interaction with biomolecules including preferential adsorption of single-stranded over double-stranded DNA, inter leaflet insertion in the hydrophobic core of lipid bilayers, DNA intercalation in the presence of copper cations, and high cargo carrying capacity for conjugated small molecule drugs, which can be physically adsorbed and reversibly desorbed (Sanchez et al. 2011). As a result, a number of biomedical applications have also been proposed for GFMs, with the largest set of studies focusing on graphene oxide in adsorption of enzyme, cell imaging, and drug delivery, as well as biosensors (Wang et al. 2011).

The purpose of this review is to compile up-to-date information pertaining to the biological and toxicological activity for GFMs. The aim of the review is to identify, summarize, and present information on the influence of GFM on bacteria, mammalian cells, animals, and plants on the basis of the most recent literature in the field. This article also presents the results of in vitro and in vivo tests and potential mechanisms of toxicity. Moreover, this article studies the effect of functionalization of GFMs on pacifying their strong interactions with cells and related toxic effects. A formal literature search. The article discusses the results of a formal literature review which was conducted using several international databases of scientific papers such as Science Direct, Web of Science, PubMed-NCBI, and Scirus. It has to be noted that in the case of manuscripts as well as tables and figures the use of the original GFMs names has been pertained. Such an approach will facilitate a detailed comparison of GFM properties.

Another aim of this review is to provide appropriate information to the scientific community so that it can be used to conduct an exposure assessment and evaluate the environmental and human health toxicity of GFMs as they are manufactured and will be introduced into the domestic market and, subsequently, the environment.

## Toxicity

### Toxicity to bacteria

Some studies on bacterial toxicity of GFM suggest that these materials could be used in antimicrobial products (Akhavan 2010; Hu et al. 2010). Bacterial toxicity of these materials was investigated, so far, for both Gram-positive and Gram-negative bacteria (Table 1).

**Table 1:** Summary of the graphene family materials toxicity toward bacteria species

Original GFM name	Bacterially-reduced graphene oxide sheets	Graphene oxide sheets	Reduced graphene oxide	Graphene oxide	Graphite oxide	Graphite	Graphene
Properties (measurement method)	No data	~1 nm TH, multi-layered, smooth planar structure (AFM)	2.75 ± 1.18 μm LD (SEM)	1 nm TH, single-layered (AFM) 0.31 ± 0.20 μm LD (SEM)	6.28 ± 2.50 μm LD (SEM)	6.87 ± 3.12 μm LD (SEM)	No data
Surface modifications	No modifications	No modifications	No modifications	No modifications	No modifications	No modifications	No modifications
Method of synthesis	Graphene oxide reduction by *E. coli* species	Natural graphite powder oxidization by modified Hummers method	Graphene oxide reduction by hydrazine	Graphite oxide powder bath sonication in water at 550 W for 6 h	Graphite powder oxidization by modified Hummers method	Graphite powder sonication in isotonic saline solution using a bath sonicator	Graphene oxide reduction by *Shewanella* species
Investigated bacteria	*E. coli*	*E. coli*	*E. coli*	*E. coli*	*E. coli*	*E. coli*	*Shewanella*
Toxicity investigations results (measurement method)	Slight toxicity (spread plate method and metabolic activity ATP test)	No toxicity (spread plate method and metabolic activity ATP test)	~45.9 % viability loss (spread plate method)	~69.3 % viability loss (spread plate method)	~15.0 % viability loss (spread plate method)	~26.1 % viability loss (spread plate method)	No toxicity (Spectrophotometry)
Toxicity mechanism (measurement method)	Inhibition of cells proliferation (XPS)	Acted as biocompatible sites for adsorption and proliferation of cells (XPS)	Loss of cells integrity, cells were individually wrapped by nanosheets (SEM) 94.2 ± 1.1 % glutathione loss (glutathione oxidation assay) ROS-independent oxidative stress (XTT assay)	Loss of cells integrity, cells were individually wrapped by nanosheets (SEM) 22.2 ± 0.7 % glutathione loss (glutathione oxidation assay) ROS-independent oxidative stress (XTT assay)	21.4 ± 1.1 % glutathione loss (glutathione oxidation assay) ROS-independent oxidative stress (XTT assay)	29.9 ± 0.7 % glutathione loss (glutathione oxidation assay) ROS-independent oxidative stress (XTT assay)	No toxicity
Reference	(Akhavan and Ghaderia 2012)	(Akhavan and Ghaderia 2012)	(Liu et al. 2011)	(Liu et al. 2011)	(Liu et al. 2011)	(Liu et al. 2011)	(Wang et al. 2011)

Original GFM name	Graphene oxide	Graphene	Reduced graphene oxide nanosheets	Graphene oxide nanosheets	Reduced graphene nanowalls	Graphene oxide nanowalls
Properties (measurement method)	No data	Few µm LD, curled nanosheets (SEM) 264 m^2^ g^−1^ surface area (BET)	~1.0 nm TH, single-layered (AFM) from nm to µm LD, curled nanosheets (SEM)	~1.1 nm TH, single-layered (AFM) from nm to µm LD, curled nanosheets (SEM)	~500 nm LD with random orientations versus substrate, very sharp edges, curled nanosheets	~500 nm LD with random orientations versus substrate (SEM)
Surface modifications	No modifications	No modifications	No modifications	No modifications	No modifications	No modifications
Method of synthesis	Graphite powder oxidization by modified Hummers method	Graphene oxide reduction by hydrazine hydrate	Graphene oxide reduction by hydrazine hydrate	Graphite powder oxidization by modified Hummers method	Graphene oxide nanowalls film (on stainless steel substrate) reduction by hydrazine	Graphene oxide synthesis by a chemical exfoliation method and graphene oxide nanowalls film deposition on stainless steel substrate
Investigated bacteria	*Shewanella*	*E. coli*	*E. coli*	*E. coli*	*E. coli*, *S. aureus*	*E. coli*, *S. aureus*
Toxicity investigations results (measurement method)	No toxicity (Spectrophotometry)	No toxicity (SEM)	24 % metabolic activity reduction at 85 µg ml^−1^ (metabolitic activity test via luciferase-based ATP assay) 90 % viability loss (spread plate method)	13 % metabolic activity reduction at 85 µg ml^−1^ (metabolitic activity test via luciferase-based ATP assay) 98.5 % viability loss (spread plate method)	*E. coli* *→* *→* ~84 % viability loss, after 1 h *S. aureus* *→* *→* ~ 95 % viability loss, after 1 h (colony counting method)	*E. coli* *→* ~59 % viability loss, after 1 h *S. aureus→* ~74 % viability loss, after 1 h (colony counting method)
Toxicity mechanism (measurement method)	No data	No data	Cell membrane damage due to contact interaction (TEM)	Cell membrane damage due to contact interaction (TEM)	Cell membrane damage due to charge transfer (RNA concentration measurement)	Cell membrane damage due to charge transfer (RNA concentration measurement)
Reference	(Wang et al. 2011)	(Zhang et al.^ 2011^)	(Hu et al. 2010)	(Hu et al. 2010)	(Akhavan and Ghaderi 2010)	(Akhavan and Ghaderi 2010)

*LD* lateral dimensions, *TH* thickness, *XTT* 2,3-bis (2-methoxy-4-nitro-5-sulfophenyl)-2*H*-tetrazolium-5-carboxanilide, *ATP* adenosine triphosphate, *ROS* reactive oxygen species, *SEM* scanning electron microscope, *TEM* transmission electron microscope, *AFM* atomic force microscope, *XPS* X-ray photoelectron spectroscope

Akhayan et al. (2010) researched the antibacterial activity of both graphene oxide and reduced graphene on Gram-negative *Escherichia coli*, and Gram-positive *Staphylococcus aureus* strains. The results showed that the graphene oxide reduced by hydrazine was more toxic to the bacteria than the unreduced graphene oxide. Moreover, better antibacterial activity of the reduced graphene was assigned to better charge transfer between the bacteria and more sharpened edges of the reduced graphene, during contact interaction. On the basis of measuring the efflux of cytoplasm of the bacteria, authors found that cell membrane damage of the bacteria caused by direct contact of the bacteria with the extremely sharp edges of nanowalls was an effective mechanism in bacterial inactivation. However, *E. coli* bacteria with an outer membrane were more resistant to the cell membrane damage than the *S. aureus* lacking the outer membrane. Similar results were obtained by Hu et al. (2010). They reported the antibacterial activity of graphene oxide and reduced graphene oxide only on *E. coli*. Within 2 h, *E. coli* cell metabolic activity was reduced to approximately 70 and 13 % at concentrations of 20 and 85 μg ml^−1^, respectively. Authors also confirmed that graphene and graphene oxide produce bacterial membrane damage upon contact and caused loosing its membrane integrity.

Liu et al. (2011) compared the antibacterial activity of four types of GFMs, namely graphite, graphite oxide, graphene oxide, and reduced graphene oxide toward *E. coli*. The results indicated that under similar concentration (40 μg ml^−1^), graphene oxide dispersion showed the highest antibacterial activity, sequentially followed by reduced graphene oxide, graphite, and graphite oxide. SEM images suggested that cell direct contact with graphene nanosheets disrupted the cell membrane. However, no reactive oxygen species (ROS) production was detected together with the glutathione oxidization ability. The authors also concluded that antimicrobial actions resulted from both membrane and oxidation stress. Therefore, the researchers proposed a three-step antimicrobial mechanism, including: initial cell deposition on graphene-based materials, membrane stress caused by direct contact with sharp nanosheets, and the ROS-independent oxidation stress.

In comparison, three studies reported lack of graphene 41 (Zhang et al. 2011) and graphene oxide toxicity to bacteria (Wang et al. 2011; Akhavan 2012). Zhang et al. (2011) investigated graphene with a Brunauer–Emmett–Teller (BET) specific surface area of 264 m^2^ g^−1^ as an anodic catalyst of microbial fuel cells based on *E. coli*. Authors have found that lots of *E. coli* cells accumulated on the electrode surface and successfully adhered to one another with no inhibition of bacterial growth.

Wang et al. (2011) noted the lack of toxicity of graphene oxide to *Shewanella* species. Moreover, the graphene oxide could be reduced to graphene in a normal aerobic setup under ambient conditions as mediated by microbial respiration of *Shewanella* bacterial cells. *Shewanella* species represent an important family of dissimilatory metal-reducing bacteria, which can transfer metabolically generated electrons from a cell interior to external electron acceptors, such as solid metal oxides during anaerobic respiration. Extracellular electron transfer pathways at the cell/graphene oxide interface were systematically investigated by the authors, suggesting that both direct electron transfer and electron mediators are involved in the graphene oxide reduction.

In a very recent study, Akhavan et al. (2012) examined interactions of chemically exfoliated graphene oxide nanosheets and *E. coli* species living in mixed-acid fermentation environment and anaerobic conditions. By an XPS method, authors found that *E. coli* reduced graphene oxide to bacterially reduced graphene in a self-limiting manner. Graphene oxide sheets acted as biocompatible sites for adsorption and proliferation of bacteria cells on their surfaces, while the bacterially reduced graphene oxide sheets showed an inhibition for proliferation of the bacteria on their surfaces. Authors showed that the slight antibacterial property of the bacterially reduced graphene oxide sheets and the detaching of the already proliferated bacteria from their surface contributed to the growth inhibition of the bacteria on the surface of the reduced sheets.

### In vitro toxicity

The interaction between dispersed GFM has been studied in vitro using human cell cultures, such as fibroblasts (Wang 2011), epithelial cells (Chang et al. 2011), alveolar basal epithelial cells (Hu et al. 2011), pheochromocytoma cells, oligodendroglia cells, fetal osteoblasts (Agarwal et al. 2010), cervical cells (Gollavelli and Ling 2012), skin fibroblasts (Liao et al. 2011), red blood cells (Liao et al. 2011), epithelial breast cancer cells (Robinson et al. 2011) as well as neuronal cells (Zhang et al. 2010). Mouse neuronal (Li et al. 2011) and pheochromocytoma cells (Agarwal et al. 2010) were also analyzed and the available in vitro toxicity literature data has been summarized in Table 2.

**Table 2:** Summary of the graphene family materials in vitro toxicity

Original GFM name	Layered graphene nanoplatelets	Graphene oxide films	Reduced nanographene oxide sheets	Nanographene oxide sheets	Graphene	Graphene oxide	Graphene	Graphene oxide	Multi-function magnetic graphene
Properties (measurement method)	5.64 ± 4.56 μm LD, 1–10 layered (SEM) ~100 m^2 ^g^−1^ surface area, ~2 g cm^−3^ density (specification sheet)	No data	5–100 nm LD (AFM)	~5 μm LD (AFM)	3.018 ± 36 nm hydrodynamic diameter, −37.2 ± 1.6 mV zeta potential (Zetasizer)	Tens of nm TH , 10 μm LD (AFM) 765 ± 19 nm hydrodynamic diameter, −40.6 ± 2.9 mV zeta potential (Zetasizer)	3.018 ± 36 nm hydrodynamic diameter, −37.2 ± 1.6 mV zeta potential (Zetasizer)	Tens of nm TH, 10 μm LD (AFM) 765 ± 19 nm hydrodynamic diameter, −40.6 ± 2.9 mV zeta potential (Zetasizer)	~4–6 nm TH, 40–60 nm LD (AFM)
Surface modifications	No modifications	No modifications	Functionalized noncovalently by PEG	No modifications	No modifications	No modifications	No modifications	No modifications	1.5 ml PAA and FMA-coated 50 mg of magnetic graphene
Method of synthesis	Purchased in Cheaptubes.com	Sterile PVDF paper filters were coated with graphene oxide suspensions (75 μg per filter)	Graphene oxide was added with 1,000 mg l^−1^ of the C18-PMH-mPEG_5000_ polymer and chemically reduced by hydrazine	Graphite oxidization by modified Hummers method	Graphene oxide reduction by a simple hydrazine-free hydrothermal route	Graphite oxidization by modified Hummers method	Graphene oxide reduction by a simple hydrazine-free hydrothermal route	Graphite oxidization by modified Hummers method	Magnetic graphene was synthesized by in-situ microwave-assisted reduction and magnetization process. Then, magnetic graphene was covalently modified with PAA and FMA via sonication followed by microwave irradiation method
Investigated cell culture line	Immortalized human monocytic cell line THP-1	Mammalian colorectal adenocarcinoma HT-29 cells	MCF7 human epithelial breast cancer cells	MCF7 human epithelial breast cancer cells	Human skin fibroblast cells (CRL-2522)	Human skin fibroblast cells (CRL-2522)	Red blood cells obtained from human whole blood samples stabilized EDTA	Red blood cells obtained from human whole blood samples stabilized EDTA	Human cervical cancer (HeLa) cells
Toxicity investigations results (measurement method)	Graphene nanoplatelets was not fully phagocytosed by THP-1 cells, frustrated phagocytosis occurred (SEM) LDH release was significantly increased at >5 μg cm^−2^ (LDH membrane integrity assay)	Graphene oxide promotes mammalian cell attachment and proliferation (cell attachment assessment by light microscopy)	>50 % decrease in metabolic activity at 80.28 ± 17.28 mg l^−1^ (MTS viability assay)	>50 % decrease in metabolic activity at 99.62 ± 17.08 mg l^−1^ (MTS viability assay)	Only few % decrease in metabolic activity 200 μg ml^−1^ (MTT viability assay) >80 % decrease in metabolic activity 200 μg ml^−1^ (WST-8 viability assay)	Only few % decrease in metabolic activity 200 μg ml^−1^ (MTT viability assay) >20 % decrease in metabolic activity 200 μg ml^−1^ (WST-8 viability assay)	50 % of the blood cells were hemolysed at >200 μg ml^−1^ (hemolysis assay)	50 % of the blood cells were hemolysed at 142 μg ml^−1^ (hemolysis assay)	<60 % decrease in metabolic activity at 200 μg ml^−1^ (MTT viability assay) ~225 % LDH release at 200 μg ml^−1^ (LDH membrane integrity assay)
Toxicity mechanism (measurement method)	Loss of membrane integrity could be due to generation of ROS (TEM)	No data	No data	No data	Concentration-dependent generation of ROS in cells (ROS assay)	Concentration-dependent generation of ROS in cells (ROS assay)	No data	No data	Low ROS generation and cell apoptosis. Excellent co-localization in the cytoplasmic region without any surface agonist (ROS assay)
Reference	(Schinwald et al. 2012)	(Ruiz et al. 2011)	(Robinson et al. 2011)	(Robinson et al. 2011)	(Liao et al. 2011)	(Liao et al. 2011)	(Liao et al. 2011)	(Liao et al. 2011)	(Gollavelli and Ling 2012)

Original GFM name	PAA-grafted magnetic graphene	Graphene oxide	Reduced graphene oxide film	Graphene film	Graphene sheets	Graphene oxide nanosheets	Graphene oxide nanosheets	Graphene oxide	Graphene oxide
Properties (measurement method)	No data	1–3 nm TH, 40–60 nm LD, 1–3 layered (AFM)	1.6 nm surface roughness (AFM)	3–5 layered (micro-Ramann, TEM) 4.49 ± 0.09 nm surface roughness (AFM) ~90 % optical transmittance (UV–Vis spectroscopy)	3–5 nm TH, 100–110 nm LD, 3–5 layered (TEM, AFM, XRD) −35.59 mV zeta potential (Zeta-reader)	~1.0 nm TH, single-layered (AFM)	~1.0 nm TH, single-layered (AFM)	Thickness ~0.9 nm, single or few layers, lateral dimensions from 160 to 780 nm (TEM, AFM)	~1 nm TH, ~few μm LD, single-layered, flat and smooth sheets (TEM, AFM)
Surface modifications	1.5 ml PAA-coated 50 mg of magnetic graphene	No modifications	No modifications	0.1 mg ml^−1^ PLL-coated graphene oxide	No modifications	20 μg ml^−1^ FBS-coated graphene oxide	No modifications	No modifications	No modifications
Method of synthesis	Magnetic graphene was synthesized by in-situ microwave-assisted reduction and magnetization process. Then, magnetic graphene was covalently modified with PAA via sonication followed by microwave irradiation method	Pristine graphite flakes oxidization by modified Hummers method	Dispersed graphene oxide in methanol was spin-coated onto a cleaned hydrophilic Si/SiO_2_ wafer and then chemically reduced by hydrazine. The resulting reduced graphene oxide film was detached using NaOH and transferred onto coverslips and dried in the air	Chemical vapor deposition (CVD) method	Synthesis using radio frequency catalytic (Fe–Co/MgO, 2.5:2.5:95 wt%) chemical vapor deposition technique	Natural graphite powder oxidization by modified Hummers method and coating with FBS	Natural graphite powder oxidization by modified Hummers method	Natural graphite powder oxidization by modified Hummers method	Natural graphite powder oxidization by modified Hummers method
Investigated cell culture line	Human cervical cancer (HeLa) cells	Human cervical cancer (HeLa) cells	Mouse pheochromocytoma (PC12) cells Human oligodendroglia (HOG) cells Human fetal osteoblast (hFOB) cells	Mouse neuronal cells delivered from the brain of postnatal 1 day old ICR mouse	Human neuronal PC12 cells	Adenocarcinomic human alveolar basal epithelial cells (A549 cell line)	Adenocarcinomic human alveolar basal epithelial cells (A549 cell line)	Human lung epithelial cells (A549 cell line)	Human lung fibroblasts
Toxicity investigations results (measurement method)	<60 % decrease in metabolic activity at 200 μg ml^−1^ (MTT viability assay) ~130 % LDH release at 200 μg ml^−1^ (LDH membrane integrity assay)	<60 % decrease in metabolic activity at 200 μg ml^−1^ (MTT viability assay) ~80 % LDH release at 200 μg ml^−1^ (LDH membrane integrity assay)	Good biocompatibility with all three cell types (MTT viability assay)	Excellent biocompatibility, cell viability, and morphology were not affected. Neurite numbers and average neurite length on graphene substrate were significantly enhanced (MTT viability assay and LDH membrane integrity assay)	Concentration and shape-dependent cytotoxicity and mitochondrial injury after 4 and 24 h at 10 μg ml^−1^ (MTT assay) High LDH release at 100 μg ml^−1^ (LDH membrane integrity assay)	0 % viability loss (MTT viability assay)	~50 % decrease in metabolic activity at 100 μg ml^−1^ (MTT viability assay)	~1.5 % viability loss at 200 μg ml^−1^ (Trypan blue exclusion assay). <20 % viability loss (CCK-8 viability assay) 1.1–2.4 % apoptosis rate at 200 μg ml^−1^ (FITC Annexin V Apoptosis Detection Kit I) ~6 % LDH leakage at 200 μg ml^−1^ (LDH membrane integrity test-kit)	<20 % viability loss at <20 μg ml^−1^ >40 % viability loss at >50 μg ml^−1^ (CCK-8 assay)
Toxicity mechanism (measurement method)	No data	Dose-dependent oxidative stress was observed at higher concentrations (No data)	No data	Graphene promoted neurite sprouting and outgrowth to the maximal extent. GAP-43 Protein expression was greatly enhanced (GAP-43 expression assay)	Concentration- and time-dependent ROS generation (ROS detection via oxidant-sensitive dye DCFH-DA) Time-dependent caspase 3 activation indicated a weak caspase 3-mediated apoptotic pathway (Caspase 3/7 assay)	No cell membrane damage (TEM)	Destroys cell membranes and directly induces cell death (TEM) Serum-mitigated cytotoxicity due to high protein adsorption ability (AFM)	Hardly entered cells and showed good biocompatibility. Oxidative stress was also detected. ROS level was 3.9 times of control at 200 μg ml^−1^ (ROS detection via oxidant-sensitive dye DCFH-DA)	Entered into cells cytoplasm and nucleus, decreased cell adhesion, induced cell floating and apoptosis (TEM)
Reference	(Gollavelli and Ling 2012)	(Gollavelli and Ling 2012)	(Agarwal et al. 2010)	(Li et al. 2011)	(Zhang et al. 2010)	(Hu et al. 2011)	(Hu et al. 2011)	(Chang et al. 2011)	(Wang et al. 2011)

*LD* lateral dimensions, *TH* thickness, *TEM* transmission electron microscope, *AFM* atomic force microscope, *XRD* X-ray diffraction, *CCK-8* cell counting kit-8, *LDH* lactase dehydrogenase, *DCFH-DA* 2′,7′-dichlorofluorescin diacetate, *FITC* fluorescein isothiocyanate, *ROS* reactive oxygen species, *MTT* 3-(4,5-dimethylthiazol-2-yl)-2,5-diphenyltetrazolium bromide, *MTS* 3-(4,5-dimethylthiazol-2-yl)-5-(3-carboxymethoxyphenyl)-2-(4-sulfophenyl)-2*H*-tetrazolium, *WST* water-soluble tetrazolium salt, *FBS* fetal bovine serum, *GAP-43* growth associate protein-43, *PLL* poly-l-lysine which is commonly used to promote cell adhesion and proliferation, *PAA* polylactic acid, *FMA* fluorescein o-methacrylate, *PEG* polyethylene glycol

Literature data indicate that exposure to GFM may induce severe cytotoxicity and lung diseases. Wang et al. (2011) demonstrated that graphene oxide could produce cytotoxicity in dose- and time-dependent means, and can enter human lung fibroblasts cytoplasm and nucleus, decreasing cell adhesion, and inducing cell floating and apoptosis at doses above 20 μg ml^−1^ after 24 h. The results indicated that graphene oxide of dose less than 20 μg ml^−1^ failed to exhibit toxicity to human fibroblast cells, while the dose of more than 50 μg ml^−1^ exhibited obvious cytotoxicity reflected in decreasing cell adhesion or inducing cell apoptosis during 1–5 days following cell seeding. Authors also confirmed that GFM can enter the lung tissues and stop there and induce lung inflammation and subsequent granulomas highly dependent on injected dose.

Chang et al. (2011) also investigated toxicity of graphene oxide by examining its influence on the morphology, viability, mortality, and membrane integrity of human lung epithelial cells. However, the results suggested that graphene oxide did not enter cells and had no obvious cytotoxicity. Authors found out that graphene oxide could only cause a slight dose-dependent oxidative stress in cell and induce a slight loss of cell viability even at the concentration of 50 μg ml^−1^.

Hu et al. (2011) have also carried out a systematic study on cellular effects of graphene oxide. Authors observed that human alveolar basal epithelial cells (A549) were sensitive to the presence of graphene oxide and showed concentration-dependent cytotoxicity.

Zhang et al. (2010) proved that graphene could induce cytotoxic effects and mitochondrial injury in human neuronal cells after 4 and 24 h at a dose of 10 μg ml^−1^. The effects observed in the examination were concentration- and shape-dependent. Interestingly, at low concentrations, graphene induced stronger metabolic activity than carbon nanotubes, and this trend was, however, reversed at higher concentrations. Furthermore, time-dependent caspase 3 activation after exposure to graphene (10 μg ml^−1^) showed the evidence of neuronal cells apoptosis. Gollavelli and Ling (2012) studied in vitro cytotoxicity of graphene to human cervical cancer cells (HeLa). The results suggested that graphene exhibited toxicity with an IC50 value of ~100 mg ml^−1^.

Liao et al. (2011) showed that the toxicity of graphene and graphene oxide depends on the exposure environment (i.e., whether or not GFM aggregation occurs) and mode of interaction with cells. Authors explored the toxicity of GFM toward human red blood cells and skin fibroblasts. The greatest hemolytic activity was displayed by the graphene oxide, whereas aggregated graphene sheets exhibited the lowest hemolytic properties. Water-soluble tetrazolium salt (WST-8), trypan blue exclusion and ROS assays revealed that the graphene sheets were more damaging to mammalian fibroblasts than the graphene oxide and generated significant amount of ROS in human skin fibroblast cells. These GFMs also strongly bound to the cell surface.

Schinwald et al. (2012) also demonstrated that the layered (1–10 layers) graphene nanoplatelets exceeding a size of approximately 15 μm projected diameter could not be fully phagocytosed by immortalized human monocytic (THP-1) cells which led to inhibition of phagocytosis process and frustrated phagocytosis occurrence. Authors also found that concentrations of 5 μg cm^−2^ and higher significantly increased the LDH release resulting in loss of membrane integrity and decrease in macrophages viability. The loss of membrane integrity could be a result of generation of ROS.

In comparison with these studies, some results have shown that GFMs in the form of film can exhibit excellent biocompatibility with no viability inhibition of investigated cells.

Reduced graphene oxide in the form of a film was found to be non-toxic to the cells examined. Agarwal et al. (2010) studied the ability of reduced graphene oxide films in inducing toxic effects in three types of cells, such as mouse pheochromocytoma cells, human oligodendroglia cells, and human fetal osteoblasts. The authors found that reduced graphene oxide showed good biocompatibility with all these cell types.

Ruiz et al. (2011) also studied the role of graphene oxide film (glass slides coated with 10 μg of graphene oxide) on mammalian colorectal adenocarcinoma HT-29 cells attachment and proliferation using light microscopy. The results indicated that the mammalian cells were attached more efficiently to the graphene oxide films with no damage on cells morphology or enlargement. These results clearly showed that the graphene oxide films exhibited no toxicity to the investigated cells and actually promoted their attachment and proliferation.

Similar results were obtained by Li et al. (2011). Authors observed good biocompatibility of graphene films toward mouse neuronal cells. Authors observed that cells numbers and average neurite length on graphene films were significantly enhanced during 2–7 days following cell seeding. These results suggested that graphene could efficiently promote neuronal cells growth. However, it should be noted that these films were additionally coated with PLL which makes these results difficult to compare with other results.

### In vivo toxicity

Only five studies reported biodistribution and toxicity of graphene oxide following intravenous and intratracheal injection in mice (Table 3). Wang et al. (2011) divided thirty Kun Ming mice into three test groups (low, middle, high dose) and one control group. Test groups were injected intravenously with 0.1, 0.25, and 0.4 mg graphene oxides, respectively. Graphene oxide under low dose (0.1 mg) and middle dose (0.25 mg) did not exhibit visible toxicity to mice and under high dose (0.4 mg) exhibited chronic toxicity (4 out of 9 mice died). At a dose of 0.4 mg graphene oxide caused granuloma formation, in the kidneys, lungs, liver, spleen, and could not be cleaned by kidney. At a dose of 0.4 mg graphene oxide was not filtrated by the kidneys.

Similar results were obtained by Zhang et al. (2011) who investigated the distribution and biocompatibility of graphene oxide in Kun Ming mice. The use of radiotracer technique revealed high uptake and long term retention of graphene oxide in the lungs as well as a relatively long blood circulation time. No significant pathological changes in all the examined organs were observed following the exposure to 1 mg kg^−1^ of graphene oxide for 14 days. However, 10-fold increase of the dose led to forming significant pathological changes. Following the exposure to 10 mg kg^−1^ body weight of graphene oxide for 14 days, authors observed significant pathological changes, such as inflammation, cell infiltration, pulmonary edema, and granuloma formation in the lungs of mice.

**Table 3:** Summary of the graphene family materials in vivo toxicity

Original GFM name	Graphite nanoplatelets	Multi-function magnetic graphene	Graphene oxide	Graphene	Graphene	Graphene oxide	Graphene oxide
Properties (measurement method)	1–20 nm TH, from 1–10 of μm LD, 3–60 layered (SEM)	~4–6 nm TH, 40–60 nm LD (AFM)	0.5–2.0 nm TH (AFM)	1.2–5.0 nm TH (AFM)	1.2–5.0 nm TH (AFM)	~1 nm TH, 10–800 nm LD, single-layered (AFM)	~1 nm TH, few μm LD, single-layered, flat and smooth sheets (TEM, AFM)
Surface modifications	No modifications	1.5 ml PAA and FMA-coated 50 mg of magnetic graphene	No modifications	Graphene was dispersed by 2 % Pluronic (block copolymer)	No modifications	Radiolabeled with ^188^Re	No modifications
Method of synthesis	No data	Magnetic graphene was synthesized by in-situ microwave-assisted reduction and magnetization process. Then, magnetic graphene was covalently modified with PAA and FMA via sonication followed by microwave irradiation method microwave-heated sonication-assisted process	Natural graphite oxidization by modified Hummers method	Ultrasonication of natural graphite flakes	Ultrasonication of natural graphite flakes	Graphite powder oxidization by modified Hummers method	Natural graphite powder oxidization by modified Hummers method
Investigated animal (injection pathway)	*Caenorhabditis elegans* nematodes (No data)	Wild-type AB strains of* Danio rerio* (zebrafish) embryos (10 nl volume was microinjected into the zebrafish pole region of embryos)	C57BL/6 mice, male, 20–25 g, 8–12 weeks old (50 μg/mouse intratracheal injection pathway—direct administration into lungs)	C57BL/6 mice, male, 20–25 g, 8–12 weeks old (50 μg/mouse intratracheal injection pathway—direct administration into lungs)	C57BL/6 mice, male, 20–25 g, 8–12 weeks old (50 μg/mouse intratracheal injection pathway—direct administration into lungs)	Kun Ming mice, male, 20 ± 2 g, 6–8 weeks old (intravenous injection pathway)	Kun Ming mice, female, 28–30 g, 4–5 weeks old (intravenous injection pathway)
Toxicity investigations results (measurement method)	No acute or chronic toxicity (longevity measurement) No effect on reproductivity (reproductivity assessment)	No influence on survival rate	Lung injury, severe inflammation with alveolar exudates, hyaline membrane formation, leakage of protein into the alveolar space (low power electron micrography)	Homogeneous distribution in lungs, minimal histologic evidence of lung inflammation (low power electron micrography)	Nonhomogeneous distribution in lungs, minimal histologic evidence of lung inflammation (low power electron micrography)	No data on mortality because all mice were artificially sacrificed after graphene oxide injection	No mortality with <0,25 mg dose, 50 % mortality with 0.4 mg dose (mortality assessment after 1–7 days)
Toxicity mechanism (measurement method)	Good distribution along the whole body, transition from the intestine to the gonads (FT-IR mapping)	Biocompatible, good distribution from the head to tail (whole-animal microscopic imaging)	No data	No data	No data	Cell infiltration and significant pathological changes: inflammation, pulmonary edema, granuloma formation in the lung when exposed to 10 mg kg^−1^ body weight for 14 days (gamma-ray radioactivity counter)	Accumulation and high granuloma formation in lungs, liver, kidney, and spleen. No brain accumulation due to blood–brain barrier (organs histopathology and light micrography)
Reference	(Zanni et al. 2012)	(Gollavelli and Ling 2012)	(Duch et al. 2011)	(Duch et al. 2011)	(Duch et al. 2011)	(Zhang et al. 2011)	(Wang et al. 2011)

*LD* lateral dimensions, *TH* thickness, *TEM* transmission electron microscope, *SEM* scanning electron microscope, *AFM* atomic force microscope, *PAA* polylactic acid, *FMA* fluorescein o-methacrylate, *FT-IR* fourier-transform infrared spectroscopy

Duch et al. (2011) administered the solutions of pristine graphene, Pluronic (block copolymer) dispersed graphene, and graphene oxide directly into the lungs of six C57BL mice. The introduction of graphene oxide resulted in severe and persistent lung injury. The examination of the lung tissues revealed an increased rate of mitochondrial respiration and the generation of ROS as well as the presence of activated inflammatory and apoptotic pathways.

As for in vivo studies on the GFM toxicity to other living organisms, Gollavelli and Ling experimented on fish (Gollavelli and Ling 2012) and Zanni experimented on nematodes (Zanni et al. 2012). Gollavelli and Ling (2012) studied in vivo cytotoxicity of GFM to *Danio rerio* (zebrafish) embryos microinjected with multi-function graphene (coated with PAA and FMA). The studies proved that this material was biocompatible with zebrafish and failed to induce any significant abnormalities or affect the survival rate of fish embryos. Confocal laser scanning microscopy images revealed that multi-function graphene was located only in the embryo’ cytoplasm region and exhibited good biodistribution from the head to tail in the zebrafish. However, it should be noted that the multi-function graphene used in the study was coated with PAA and PLL which could lead to the lack of toxicity of the material.

Zanni et al. (2012) evaluated the toxicity of graphite nanoplatelets in the model living organism such as *Caenorhabditis elegans* (nematode). The absence of any acute or chronic toxicity of GNPs was observed. The authors examined longevity (life expectancy) as well as reproductive capability end points. Moreover, no effect on *C. elegans* reproductive potential was found. Good spatial distribution of the GFM inside the nematodes was demonstrated with the use of Fourier-transform infrared spectroscopy (FT-IR) mapping.

### Potential mechanisms of toxicity

Uncertainty still prevails as to toxicity pathways for GFM. Two-dimensional graphene nanomaterials are unique in comparison with spherical nanoparticles or one-dimensional nanotubes or nanorods, and the chemical and physical determinants for their cellular interactions and biocompatibility are still under studies (Sanchez et al. 2011). Direct or indirect generation of ROS leading to oxidative stress in target cells is currently the main mechanism proposed for the toxicity of engineered nanomaterials (Oberdörster and Oberdörster 2005; Stone and Schins 2009). Some reports also indicate that generation of ROS in target cells is a potential mechanism for graphene toxicity [14]. It should be also noted that cellular homeostasis process produces a balance between the level of ROS generation and its elimination or reduction by antioxidant enzymes. The level of ROS is balanced by the action of superoxide dismutase, catalase, or glutathione peroxidase. When it cannot be reduced by cellular antioxidant activity, this may lead to alteration of macromolecules such as polyunsaturated fatty acids in membrane lipids, protein denaturation, and ultimately DNA destruction (Sanchez et al. 2011). If the level of ROS is not reduced by cellular antioxidant activity, the alteration of macromolecules such as polyunsaturated fatty acids in membrane lipids, protein denaturation, and ultimately DNA destruction may occur (Sanchez et al. 2011). Thus, the presence of extremely high hydrophobic surface areas in some GFMs may result in significant interactions with membrane lipids. This may lead in turn to direct physical toxicity or adsorption of biological molecules leading to indirect toxicity (Sanchez et al. 2011). Moreover, some studies suggest that ROS are generated in a concentration- and time-dependent manner after exposure to GFM, indicating an oxidative stress mechanism (Zhang et al. 2010).

Unfortunately, the results in the literature are inconsistent, particularly concerning the ability of graphene to enter the cells. For example, Wang et al. (2011) demonstrated that graphene oxide can enter the cytoplasm and nucleus of human lung epithelial cells or fibroblasts, decreasing cell adhesion and inducing cell floating and apoptosis. In contrast, Chang et al. (2011) reported that graphene oxide cannot enter human alveolar basal epithelial cells and has no obvious cytotoxicity.

In comparison to the studies mentioned above, Hu et al. (2011) observed that the cytotoxicity of graphene oxide which resulted from direct interactions between the cell membrane and graphene oxide led to physical damage in the cell membrane. The damage was triggered off by interactions between the cell membrane and graphene oxide nanosheets. Interestingly, the authors discovered that the cytotoxicity of graphene oxide nanosheets occurred mostly during the initial contact stage of graphene oxide and cells and was independent of exposure duration. Physical damage of the cell membrane observation, however, excludes the contribution of an oxidative stress mechanism since that is a time-dependent process. Similar results were obtained by Hu et al. in the previous studies (Hu et al. 2010). The research suggests that graphene oxide and reduced graphene oxide produced bacterial (*E. coli*) membrane damage upon direct contact. The authors confirmed these results using transmission electron microscopy, which revealed that the bacterial cells lost their membrane integrity.

By the close look at cellular functions at proteome level, Yuan et al. (2011) clearly identified the distinct pattern of cellular responses between graphene-treated cells. Overall 37 differentially expressed proteins involved in metabolic pathway, redox regulation, cytoskeleton formation,and cell growth were identified by the authors. On the basis of the protein profile, authors successfully identified the key enzymes involved in the redox processes regulation of the cell, and suggested that graphene did not trigger the up-regulation of the thioredoxin-peroxiredoxin system to counter the ROS stress or did not induce the apoptosis based on the protein profile. Li et al. (2012) also demonstrated that graphene induced cytotoxicity through the depletion of the mitochondrial membrane potential and the increase of intracellular ROS. The studies also suggest that graphene can trigger apoptosis by mitochondrial pathway activation. The MAPKs (JNK, ERK and p38) as well as the TGF-beta-related signaling pathways were activated in the graphene-treated cells, which in turn activated Bim and Bax, two pro-apoptotic member of Bcl-2 protein family. Consequently, the caspase 3 and its downstream effector proteins were activated and the execution of apoptosis was initiated.

### Toxicity versus functionalization

Several studies attempted to address the interactions of graphene and its derivatives with different molecules. There some evidence which proves that polymer chains, drugs, and targeting molecules can be covalently attached to the graphene surface and edge site, or polymers may be adsorbed onto the graphene surface to enhance solubility and biocompatibility (Yan et al. 2011).

Hu et al. (2011) carried out a systematic study on cellular effects of graphene oxide nanosheets and identified the effect of fetal bovine serum (FBS), an often-employed component in cell culture medium, on the cytotoxicity of graphene oxide. At low concentrations of FBS (1 %), human cells were sensitive to the presence of graphene oxide and showed concentration-dependent cytotoxicity. However, the cytotoxicity of graphene oxide was greatly mitigated at 10 % FBS, the concentration usually employed in cell medium.

Polyethylene glycol (PEG) conjugation to graphene oxide was examined by several authors. Yang et al. (2011) used nanographene oxide sheets coated with PEG and labeled with radioactive iodine to assess biodistribution and excretion in mice following intravenous injection. These PEG-coated graphene sheets accumulated initially in the liver, and spleen of the mice followed by gradual clearance after 3–5 days. After 3 months, the nanographene sheets were cleared and induced no toxicity at a dose of 20 mg kg^−1^. PEG-coated nanographene oxide sheets were also prepared by Sun et al. (2008) in order to impart solubility and compatibility of graphene oxide in biological environment. Authors obtained separated PEGylated graphene oxide sheets that selectively recognized and bound to B cell lymphoma cells and were soluble in buffers and serum without agglomeration. Moreover, graphene oxide sheets were found to be photoluminescent in the visible and infrared regions. Another study by Liu et al. (2008) showed that graphene is a novel class of material promising for biological applications including future in vivo cancer treatment with various aromatic, low-solubility drugs. Authors functionalized nanographene oxide with branched PEG to obtain a biocompatible graphene oxide-PEG conjugate stable in various biological solutions, and used them for attaching hydrophobic aromatic molecules including a camptothecin analog, SN38, noncovalently via π–π stacking. The resulting graphene oxide-PEG-SN38 complex exhibited excellent water solubility while maintaining its high in vitro human colon cancer cells killing potency similar to that of the free SN38 molecules in organic solvents. The efficacy of new complex GFM was far higher than that of irinotecan, a FDA-approved water-soluble SN38 prodrug used for the treatment of colon cancer.

The biocompatibility of the functionalized graphene oxide and reduced graphene oxide was analyzed along with the potential biological effects of the used dispersants in L929 mouse fibroblasts by Wojtoniszak et al. (2012). Authors investigated PEG, PEG–polypropylene, glycol–PEG (Pluronic P123), and sodium deoxycholate (DOC) as the dispersants. On the basis on the results of the study, it is possible to conclude that the toxicity depends on the type of dispersant and concentration of the nanomaterials in the suspensions. The best biological properties were observed for graphene oxide functionalized with PEG whereas the other dispersants, i.e., Pluronic 123 and DOC, produced less favorable results. The research indicates that similar to graphene oxide, reduced graphene oxide in PEG is the most biocompatible. The comparison between reduced graphene oxide and graphene oxide shows that the latter has better biocompatibility, especially at higher concentrations such as 50 and 100 g ml^−1^. Robinson et al. (2011) tested reduced graphene oxide sheets (with ~20 nm in average lateral dimension) with high near-infrared (NIR) light absorbance for potential photothermal therapy. The single-layered reduced graphene oxide sheets were functionalized noncovalently by amphiphilic PEGylated polymer chains to render stability in biological solutions. Authors reported that the PEGylated reduced graphene oxide sheets exhibited little toxicity in vitro to human epithelial breast cancer cells at concentrations well above the doses needed for photothermal heating (>80 mg ml^−1^). Also Gollavelli and Ling (2012) studied in vitro cytotoxicity of multi-function magnetic graphene (coated with PAA and FMA) to human cervical cancer cells (HeLa). Author noted that this form of graphene was non-cytotoxic and did not induce significant amounts of ROS and apoptosis in HeLa cells. In vitro cellular imaging of Multi-function magnetic graphene in HeLa cells revealed sheets localization in the cytoplasmic region of cells without any surface agonist.

Sasidharan et al. (2011) observed the effect of carboxyl functionalization of graphene in pacifying its strong hydrophobic interaction with monkey renal cells and associated toxic effects. Graphene accumulated on the cell membrane causing high oxidative stress leading to apoptosis, whereas carboxyl functionalized hydrophilic graphene was internalized by the cells without causing any toxicity.

Zhang et al. (2011) covalently conjugated graphene oxide with dextran (DEX), a biocompatible polymer widely used for surface coating of biomaterials. Graphene oxide–DEX conjugates demonstrated reduced sheet sizes, increased thickness (TH), and significantly improved stability in physiological solutions. Cellular experiments performed on human cervical cancer HeLa cells showed that DEX coating on graphene oxide remarkably reduced cellular toxicity. Graphene oxide–DEX showed obvious clearance from the mouse body after intravenous injection within a week without causing noticeable short-term toxicity to the treated animals.

### Phytotoxicity

Only one study attempted to address the interactions of graphene or its derivatives with plants. The effects of graphene on root and shoot growth, biomass, shape, cell death, and ROS of cabbage, tomato, red spinach, and lettuce, were analyzed by Begum et al. (2011). The concentrations used in the study ranged from 500 to 2,000 mg l^−1^. Combined morphological and physiological analyses indicated that after 20 days of exposure under experimental conditions, graphene significantly inhibited plant growth and biomass level. The number and size of leaves of the graphene-treated plants were reduced in a dose-dependent manner. Significant effects were also detected by authors showing a concentration-dependent increase in ROS and cell death as well as visible symptoms of necrotic lesions, indicating graphene-induced adverse effects on cabbage, tomato, and red spinach mediated by oxidative stress necrosis. Little or no significant toxic effects were observed with lettuce seedlings under the same conditions. Furthermore, authors also detected the negative effect of graphene on the morphology of roots, finding that the epidermis of the treated tomato and red spinach roots was loosely or completely detached (Begum and Fugetsu 2011).

It should be noted that the plant cell death may occur either by apoptosis or by necrosis which is considered to be the passive mechanism and may be characterized by a progressive loss of membrane integrity resulting in cytoplasmic swelling and release of cellular constituents. The above-mentioned results also indicate that the potential effect of GFM on plants may largely depend on dose, exposure time, and plant species and it deserves further attention.

## Anticancer activity

Relatively few in vitro and in vivo studies concerning GFM anticancer activity have been conducted so far. Feng et al. (2011) reported that at high concentrations (up to 300 g ml^−1^), polyethyleneimine graphene complexes significantly reduced in vitro toxicity to the treated human epithelial carcinoma (HeLa) cells. Markovic et al. (2011) carried out a comprehensive study on the photothermal anticancer activity of near-infrared (NIR)-excited graphene. The results suggest that graphene nanoparticles performed significantly better than carbon nanotubes in inducing photothermal death of human glioma (U251) cells in vitro. The mechanisms of graphene-mediated photothermal killing of cancer cells apparently involved oxidative stress and mitochondrial membrane depolarization resulting in mixed apoptotic and necrotic cell death characterized by caspase activation/DNA fragmentation and cell membrane damage, respectively. Similar results were obtained by Zhang et al. (2011). Authors demonstrated that graphene oxide modified with doxorubicin (DOX) and polyethyleneglycol (PEG) during photothermal treatment showed complete in vitro viability reduction in murine mammary tumor (line EMT6) cells as well as in vivo complete destruction of solid tumors (EMT6 tumor-bearing mice were used) without mice weight-loss or recurrence of tumors.

Yang et al. (2010) have also studied the in vivo behaviors of PEGylated nanographene sheets in tumor-bearing mice by in vivo fluorescence imaging. Each mouse was intravenously injected with PEGylated nanographene sheets (200 μl of 2 mg ml^−1^ solution for each mouse at a dose of 20 mg kg^−1^). Authors demonstrated highly efficient tumor passive targeting of graphene sheets in several different tumor models, such as murine breast cancer tumors, human epidermoid carcinoma tumors, and human glioblastoma tumors, without utilizing any targeting ligands, such as antibodies. Thus, PEGylated nanographene sheets appeared to be an excellent in vivo tumor NIR photothermal therapy agent without exhibiting noticeable toxicity to the treated mice.

Zhang et al. (2010) functionalized graphene oxide with sulfonic acid groups, which render it stable in physiological solution, followed by covalent binding of folic acid (FA) molecules to obtain a novel nanocarrier for the loading and targeted delivery of anticancer drugs such as: doxorubicin (DOX) and camptothecin (CPT), onto the FA-conjugated graphene oxide via *p*–*p* stacking. Functionalization with folic acid allowed specific targeting of the human breast cancer cells, exhibiting folic acid receptors. Authors demonstrated that FA–graphene oxide loaded with anticancer drugs showed in vitro specific targeting of cancer cells, and remarkably reduced their viability.

## Summary

There have been reported numerous studies focused on GFM biomedical applications. Some of studies regarding the bacterial toxicity of GFM suggest that they may find future application in antimicrobial products. Results suggest that the cell membrane damage of *E. coli* and *S. aure*us bacteria caused by direct contact of the bacteria with the extremely sharp edges of the nanosheets was the effective mechanism in the bacterial inactivation. However, in contrast to these studies, two studies reported lack of GFM toxicity to *E. coli* and *Shewanella* species.

Only a limited number of publications attempted to address the interactions of graphene and its derivatives with living systems. In vitro toxicity investigation suggests that GFM exhibit dose-dependent toxicity to mammalian cells (e.g., human lung fibroblasts, epithelial and alveolar epithelial cells, neuronal cells as well as red blood cells), which strongly suggests that their biocompatibility must be considered when GFMs are applied for biomedical engineering. However, only few studies reported biodistribution and toxicity of graphene oxide following intravenous injection in mice. Graphene oxide under low dose did not exhibit obvious toxicity to mice but under high dose exhibited chronic toxicity, causing significant pathological changes, such as granuloma formation, mainly located in the lungs, kidneys, liver, and spleen.

The number of published study results is also greatly limited. The results of studies indicate that GFMs in the free form (highly dispersed and no-coated) exhibit high in vitro cellular toxicity. Nevertheless, GFM in the form of film exhibited good biocompatibility with investigated cells and promoted their growth and proliferation.

Unfortunately, first alarming reports on validity of different toxicity assessment methodologies were found while compiling this review. Liao et al. (2011) discovered that the methylthiazolyldiphenyl-tetrazolium bromide (MTT) assay, a typical nanotoxicity assay failed to predict the toxicity of GFM due to the spontaneous reduction of MTT by GFM, resulting in a false positive signal. Yet, other toxicity assessment, using the water-soluble tetrazolium salt (WST-8) revealed that the investigated GFMs were highly damaging to the investigated cells resulting in acute cytotoxicity. Thus, the usage of MTT assay in predicting the cytotoxicity of GFMs needs to be very careful and other alternate toxicity assays should also be applied according to reliable MTT test results.

Little is also known about toxicity pathways for GFMs. Generation of ROS in target cells is considered to be a potential mechanism for toxicity. The extremely high hydrophobic surface area of graphene may also result in significant biomolecular and cellular interactions with membrane lipids leading to indirect toxicity. GFMs can also produce cytotoxicity in dose- and time-dependent means, decreasing cell adhesion, and inducing cell floating and apoptosis. Results indicate that graphene can induce cytotoxicity through the depletion of the mitochondrial membrane potential and the increase of intracellular ROS, and then trigger apoptosis by activation of the mitochondrial pathway. Sadly, the results in the literature are inconsistent, particularly concerning the ability of GFM to enter the cells. While some studies suggest that graphene oxide can enter the cytoplasm and nucleus of human lung epithelial cells and fibroblasts, other studies indicate that graphene oxide cannot enter human alveolar basal epithelial cells. Moreover, cytotoxicity of graphene oxide was found to occur mostly during the initial contact stage of graphene oxide and cells and was independent of exposure duration. Thus, it is possible to draw the conclusion that physical damage of the cell membrane observation excludes the contribution of an oxidative stress mechanism as that is a time-dependent process.

Several studies attempted to address the interactions of graphene or its derivatives with different molecules. There is evidence which suggests that drugs and targeting molecules can be covalently attached to the graphene surface and edge site, or polymers may be adsorbed onto the GFM surface to enhance solubility and biocompatibility. The observations of different molecules related GFM cytotoxicity effects may lead to the creation of an alternative and convenient route to engineer nanomaterials for safe biomedical and environmental applications. The biocompatibility of the functionalized graphene oxide and reduced graphene was analyzed along with the potential biological effects of the used dispersants in cells. The toxicity depended on the type of dispersant and concentration of GFM in the suspensions. Although, different polymeric substrates were used to functionalize GFM for several in vitro and in vivo studies e.g., FBS, PEG derivatives, sodium DOC and DEX, the development of biocompatible surface coating seems to be critical to engineer various functional nanomaterials for biomedical applications. Surface modification of graphene was reported to alter its toxicity, whereas graphene oxide organic conjugates were reported to reduce cellular toxicity to a remarkably higher degree than their native counterparts.

Only one study, so far, attempted to address the interactions of GFMs with plants. The effects of graphene on root and shoot growth, biomass, shape, cell death, and ROS on cabbage, tomato, red spinach, and lettuce were scrutinized. Results suggest that graphene can significantly inhibit plant growth and biomass in a dose- and time-dependent manner. The potential toxic effect of graphene also largely depends on plant species and, thus, should be given much further attention.

There are relatively few studies concerning GFM anticancer activity. GFMs appeared to be excellent in vitro and in vivo tumor NIR photothermal therapy agents. Significantly reduced viability of in vitro human glioma and human epithelial carcinoma cells was observed without exhibiting noticeable toxicity. Highly efficient tumor passive targeting of GFM has been observed in several different in vivo tumor models without utilizing any targeting ligands, such as antibodies.

**Fig. 2:**
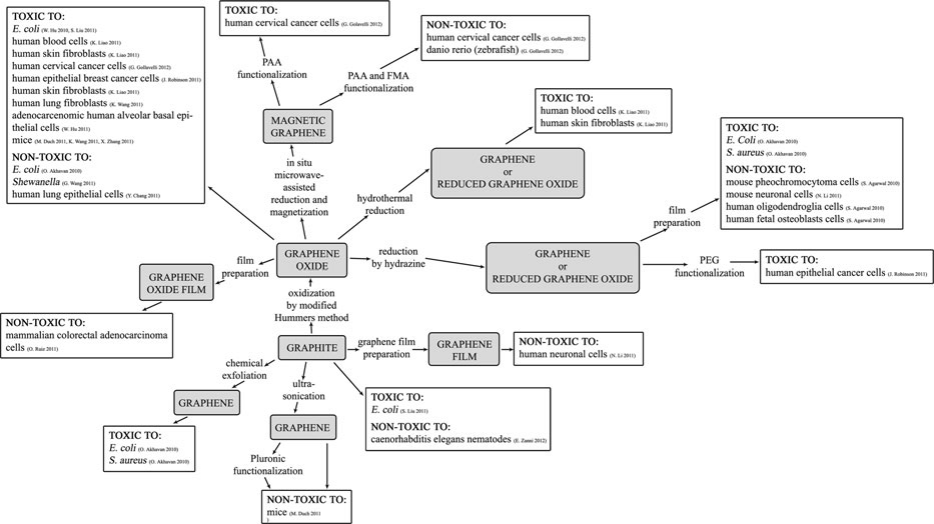
The schematic summary of the toxicological aspects of graphene family materials in relation to their synthesis techniques

**Fig. 3:**
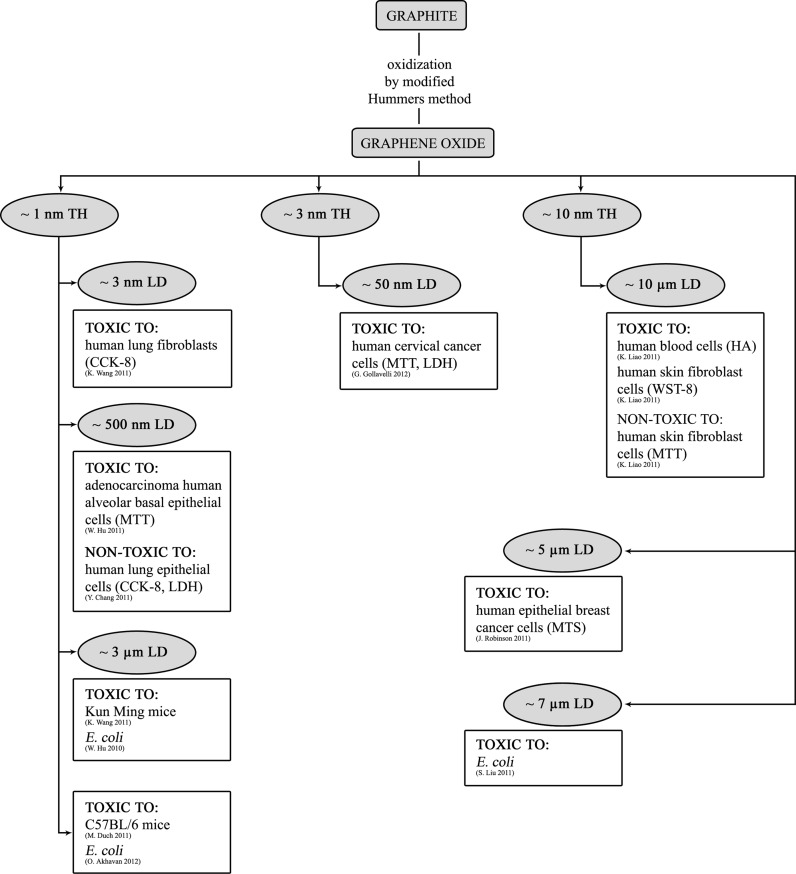
The schematic summary of the toxicological aspects of graphene oxide in relation to its synthesis techniques and chosen properties such as: thickness (TH) and lateral dimensions (LD)

What should also be taken into consideration is the fact that GFM synthesis technique determines GFM parameters and their resulting biological activity. In Figs. 2 and 3 the toxicological aspects of GFM in relation to synthesis techniques and resulting properties are presented. It is possible to notice an evident connection between the synthesis technique and bioactivity of GFM (Fig. 2). As has been mentioned before, the discrepancies in toxicological test results may be a result of different toxicity assays used and different sample preparation methods as well as differences in toxicological properties of graphene toward particular investigated cells/organisms. On the other hand, even if the same GFM preparation technique are taken into consideration, it turns out that GFMs are characterized by different properties (Fig. 3). In this article TH and lateral dimensions parameters vary the most.

To summarize, the literature on potential health risks of GFM is being published. As for toxicity, a number of studies have been conducted, yet the field still requires further research as it is a newly emerged one and the literature is still greatly limited. The sources are not sufficient to reach conclusions as to potential hazards connected with risk assessment and regulation. The most likely source of the apparent lack of uniformity are different physicochemical properties of GFMs, such as chemical structure, thickness, lateral size, surface charge, surface area, and surface modifications. Undoubtedly, these properties have significant influence on biological/toxicological activity toward investigated cells and animals. However, mentioned GFM parameters are not always well-controlled and in some cases even analyzed. Moreover, some of these parameters may also be measured by different techniques, which makes the complied results of studies almost impossible to compare. Consequently, the need for further systematic studies which would address the role of GFM parameters as well as their methods of preparation in determining adverse environmental and health impacts is not emphasized. Furthermore, some guidelines should be drawn by the community. Such guidelines would enable choosing the right GFM parameters/properties while conducting the studies. Moreover, the most applicable measurement methodologies should be recommended, and verified during scientific report submission processes.

The authors of this article believe that further work should also focus on in vitro and in vivo studies on possible mechanisms of interactions between GFMs and different living biosystems as well as decreasing GFM toxicity, which is still a great challenge for in vivo biomedical applications. Consequently, the potential impact of graphene and its derivatives (e.g., graphene oxide) on humans and environmental health need to be given the right attention. However, in order to evaluate biocompatibility of GFMs potential hazards and a systematic characterization of cellular response at protein expression level should be analyzed beforehand.
